# Mechanisms of Lung Cancer Development in Cystic Fibrosis Patients: The Role of Inflammation, Oxidative Stress, and Lung Microbiome Dysbiosis

**DOI:** 10.3390/biom15060828

**Published:** 2025-06-06

**Authors:** Raffaella Pagliaro, Filippo Scialò, Angela Schiattarella, Roberta Cianci, Susan F. M. Campbell, Fabio Perrotta, Andrea Bianco, Giuseppe Castaldo

**Affiliations:** 1 Department of Translational Medical Sciences, University of Campania ‘L. Vanvitelli’, 80131 Naples, Italy; raffaella.pagliaro@studenti.unicampania.it (R.P.); angela.schiattarella1@studenti.unicampania.it (A.S.); sfmcampbell@gmail.com (S.F.M.C.); fabio.perrotta@unicampania.it (F.P.); andrea.bianco@unicampania.it (A.B.); 2 U.O.C. Clinica Pneumologica L. Vanvitelli, A.O. dei Colli, Monaldi Hospital, 80131 Naples, Italy; cianciroberta@libero.it; 3 Department of Molecular Medicine and Medical Biotechnologies, University of Naples Federico II, 80131 Naples, Italy; giuseppe.castaldo@unina.it; 4 CEINGE-Biotecnologie Avanzate Franco Salvatore, 80145 Naples, Italy

**Keywords:** cystic fibrosis, lung cancer, chronic inflammation, oxidative stress, microbiome dysbiosis

## Abstract

Cystic fibrosis (CF) is a genetic disorder caused by mutations in the CFTR gene, leading to defective ion transport and impaired function of various organs. Chronic inflammation, oxidative stress, and microbial dysbiosis are key pathological features of CF patients, contributing to disease progression, lung damage, and an increased susceptibility to infections. Emerging evidence suggests that in CF patients these factors can promote cancer development, especially lung cancer. Chronic inflammation in CF, driven by immune cell dysfunction, results in the release of pro-inflammatory cytokines and reactive oxygen species (ROSs), fostering an environment conducive to cancer initiation. Oxidative stress can amplify cellular damage and hinder airway remodeling. ROSs not only damage cellular components such as lipids, proteins, and DNA but also disrupt lung homeostasis, creating a favorable environment for cancer development. Furthermore, the lung microbiome in CF patients is often dysbiotic, with a reduced diversity and the predominance of pathogenic bacteria such as *Pseudomonas aeruginosa*, which exacerbate inflammation and may contribute to carcinogenesis. This review explores the mechanisms linking CF to lung cancer, examining the potential clinical implications of these mechanisms for early detection, monitoring, and targeted therapies for lung cancer prevention in CF patients.

## 1. Introduction

### 1.1. Overview of Cystic Fibrosis

Cystic fibrosis (CF) is a recessive genetic disorder caused by mutations in the cystic fibrosis transmembrane conductance regulator (CFTR) gene [1]. These mutations result in a malfunctioning CFTR protein, which is crucial for regulating chloride ion transport across cell membranes, a process essential for maintaining the proper fluid balance and function of various organs, especially in epithelial cells of the lung, digestive tracts, and sweat glands [2]. The CFTR gene encodes a protein that consists of 1480 amino acids and is composed of two transmembrane domains, two nucleotide-binding domains, and a regulatory domain (R domain) [3]. R domain is phosphorylated by a protein kinase (PKA) that allows a conformational change in the CFTR protein, leading to the opening of ion channels in the membrane. CFTR mutations are classified into six classes based on their effect on the CFTR protein: protein production (Class I), protein processing (Class II), gating (Class III), conduction (Class IV), insufficient protein (Class V), and reduced stability (Class VI) [4,5]. The most common mutation is ΔF508, which is included in class II and involved in the deletion of three nucleotides in the CFTR gene, leading to the loss of the amino acid phenylalanine at position 508 in the protein [6]. This mutation leads to incorrect folding of the CFTR protein, causing it to be degraded before reaching the cell surface, and thereby severely compromising chloride ion transport [7]. In contrast, other mutations such as G551D (class III) produce a CFTR protein that is correctly trafficked to the membrane but has defective channel gating, meaning it does not open properly to allow ion passage [8,9]. These differing molecular consequences translate into distinct clinical phenotypes and therapeutic responses. As a result of these consequences, CF primarily affects the respiratory system, leading to the production of thick, sticky mucus that obstructs the airways, causing frequent lung infections and reduced lung function [10,11]. Another hallmark of the disease is its impact on the digestive system, resulting in difficulties with nutrient absorption and digestive processes [12]. Despite the improvements in terms of quality of life and life expectancy, mainly due to the introduction of new medications for the treatment of the disease, recent studies have shown a potential association between CF and an increased risk of developing certain types of cancer [13]. In particular, CF is characterized by persistent inflammation and immune system imbalances, which create a specific environment that may promote cancer initiation and progression [14,15]. Some studies have shown that inflammatory molecules, immune cells, and altered immune responses play a significant role in CF-related cancers, making it important to examine these immunological aspects to fully understand the relationship between CF and cancer [16,17].

### 1.2. Lung Cancer in CF Patients

Lung cancer (LC) is the most common malignancy worldwide in terms of both incidence and mortality [17]. As treatment for CF improves, patients live longer, thereby increasing the chance of reaching an age when LC becomes more common. Although LC remains relatively rare in individuals with CF, with an estimated incidence of less than 0.1% based on data from the CF Foundation Patient Registry, emerging evidence suggests that the risk may be slightly increased in individuals with longer disease duration and exposure to additional risk factors such as tobacco smoke. Epidemiologic studies suggest that CF patients have a higher incidence of certain subtypes of LC, which are uncommon in the general population [18]. Among these, bronchial gland carcinoma is the most frequently diagnosed, followed by squamous cell carcinoma and adenocarcinoma [19]. These subtypes are more often observed in patients with a smoking history and, therefore, a cessation of smoking is essential to reduce the incidence of LC in CF patients [20]. Smoking accounts for around 85% of LC cases in the general population. However, its specific impact on LC risk in CF patients is less well-defined, although it is recognized as a significant additional risk factor for this group [21]. According to the literature, smoking may increase LC risk in CF patients by approximately 2–3 times compared to non-smoking CF patients, but the precise attribution is limited due to the rarity of LC in this group and the presence of confounding factors [22]. Furthermore, urbanization and pollution are linked to higher rates of LC, especially in populations with pre-existing lung disease. For CF patients, pre-existing lung damage may sensitize them to environmental carcinogens [23,24]. Additionally, genetic variations among different racial and ethnic groups may influence LC risk, with specific genetic polymorphisms associated with increased risk in Asian populations, which may also modulate LC risk in CF patients with similar genetic backgrounds [25]. Genetic mutations in the CFTR gene may create a cellular environment that promotes the development of LC [26,27]. However, organ-specific cancer risks may be linked to the different localization and expression of the CFTR gene, as well as the different sensitivities of individual organs. One possible explanation for the higher incidence of cancer in the digestive tract compared to the respiratory tract is due to the reduction in expression of CFTR after birth, which remains minimal in adult respiratory epithelia [28].

A recent case–control study found that the ΔF508 deletion, along with specific genetic variants, was inversely associated with LC risk, with a 68% reduced risk in individuals carrying a particular haplotype [29]. These findings suggest that CFTR genetic variations might influence LC risk, with the ΔF508 mutation potentially offering a protective effect. However, the exact mechanisms and the possible implications with other mutation remain unclear, and further research is needed. Although the risk is higher, the overall incidence of LC in CF patients is still relatively low compared to the general population. Regular screening and monitoring for LC are essential for CF patients, as early detection is key to improving prognosis and survival outcomes. In this review, we explore the underlying mechanisms contributing to the development of LC in CF patients, with a particular focus on the role of chronic inflammation, oxidative stress, and lung microbiome dysbiosis. In particular, the objectives are to understand the interactions between inflammation, oxidative stress, and microbiome dysbiosis in creating a tumor-promoting microenvironment in the lungs of CF patients and to assess the clinical implications of these mechanisms for early detection, screening strategies, and the development of targeted therapeutic approaches for LC prevention and management in CF patients.

## 2. Chronic Inflammation in Cystic Fibrosis

### 2.1. Role of Inflammation in CF Pathogenesis

Inflammation is a key factor in the pathophysiology of CF and is considered to be one of the main drivers of disease progression, especially in the lungs. This starts early in the disease process; recent evidence suggests that the CF airways are in a pro-inflammatory state, creating an environment that facilitates tissue damage and promotes the development of chronic infections [30,31]. Neutrophils are the primary type of immune cells that infiltrate the CF airways and are recruited to the site of infection, leading to the release of enzymes and pro-inflammatory molecules which cause further damage to lung tissues [32]. The chronic inflammation in CF involves a variety of other immune responses, including pro-inflammatory cytokines like IL-8, IL-6, and TNF-α, which contribute to the persistent influx of neutrophils, which release harmful substances such as reactive oxygen species (ROS) and proteolytic enzymes, leading to damage in surrounding tissues [33,34]. Increased levels of these pro-inflammatory cytokines have been shown in the sputum and bronchoalveolar lavage fluid (BALF) of CF patients and are correlated with the amplification of airway inflammation [35]. In particular, studies have shown that high levels of neutrophils and IL-8, the main chemoattractant for neutrophils, are present in the BALF of patients even in the absence of infection, indicating that the inflammation occurs before the onset of infection [36]. In addition, the release of neutrophil extracellular traps (NETs) has been shown to contribute to lung damage by promoting fibrosis [37]. Moreover, monocytes in CF patients have been found to exhibit altered DNA methylation patterns, which are linked with a hyperinflammatory state [38]. This suggests that epigenetic changes could contribute to the dysregulated immune response in CF. Specific histone modifications in macrophages are associated with an increased expression of pro-inflammatory genes [39]. A theory attracting increasing interest suggests that immune cell dysregulation plays a central role in the chronic lung inflammation seen in CF, independent of external factors such as infections or oxygen deprivation [40]. Evidence indicates that the loss of CFTR function in immune cells, such as lymphocytes, neutrophils, and macrophages, contributes to the persistent inflammation. Studies involving CFTR-deficient mice have shown that higher levels of pro-inflammatory cytokines in CF lungs result from CFTR deficiency in immune cells, particularly macrophages [41]. Furthermore, epigenetic alterations, including changes in DNA methylation and histone modifications in monocytes and macrophages, may drive a hyperinflammatory state, highlighting the role of epigenetic changes in immune dysfunction [42]. The initial inflammatory response, triggered by CFTR malfunction or altered epigenetic modifications, is further amplified by bacterial colonization [43]. Microbes proliferate in the thick mucus layer, which results from defective airway surface liquid and impaired mucociliary clearance, contributing to persistent inflammation. Over time, the inability to control this inflammatory response causes continuous tissue damage, the formation of thick inflammatory exudates, and bronchiectasis [44]. Eventually, this chronic damage leads to irreversible airway injury, progressive lung decline, and respiratory failure, which can result in fatal outcomes. The persistent inflammation in CF is a major contributor to complications such as bronchiectasis, respiratory failure, and fibrosis [45].

### 2.2. Inflammatory Mediators and Their Contribution to Cancer Risk in CF Patients

Inflammation is widely recognized as a key feature of cancer because of its involvement in several processes of tumor development, including epithelial–mesenchymal transition, cell proliferation, survival, angiogenesis, and invasion [46]. During the initiation phase, inflammatory cytokines, such as Tumor Necrosis Factor Alpha (TNFα), IL-1β, and IL-6, activate transcription factors like Nuclear Factor kappa-light-chain-enhancer of activated B cells (NF-κB) and Signal Transducer and Activator of Transcription 3 (STAT3) in cancer cells, triggering genes that regulate cell survival, proliferation, and growth [47]. Transforming Growth Factor Beta (TGF-β) acts as a dual-function cytokine in inflammation and cancer, being protective in acute injury while promoting inflammation and tissue remodeling in chronic injury and cancerous environments. In the tumor microenvironment, cancer-associated fibroblasts (CAFs) are the primary producers of TGF-β, leading to excessive extracellular matrix (ECM) deposition and the development of fibrosis [48]. Numerous studies have identified positive correlations between serum inflammatory biomarkers and cancer-related mortality, including CRP, IL-6, leukocytes, neutrophils, haptoglobin, albumin, and TNF [49]. Systemic inflammation can serve as an indicator of vulnerability in cancer patients. The balance between pro-inflammatory and anti-inflammatory signals is essential to maintaining physiological functions and preventing cancer progression. In particular, IL-10 plays a key role in modulating inflammation and supporting tumor growth by regulating cytokines such as IL-12/IL-23 and IL-6, which stimulate inflammatory T cells [50]. Moreover, IL-10 is important for the anti-inflammatory regulatory T cells (Tregs) and the suppression of IL-17-producing T cells (Th17). Chronic inflammation is also characterized by changes in miRNA profiles. Recent studies have shown the link between CFTR dysfunction and dysregulation in miRNA expression [51]. Interestingly, 11 miRNAs among the 41 dysregulated in CF display trends that mirror those observed in LC [51]. The biological processes most affected by miRNA dysregulation are the activation of Epidermal Growth Factor Receptor (EGFR) and the unfolded protein response (UPR) [52]. In particular, EGFR mutations are commonly observed in cancer and the activation of the EGFR pathway is increased in the airway epithelium of CF patients [53]. In cancer, the UPR acts as a pro-survival mechanism, playing a role in the initiation and progression of several types of cancer. The F508del-CFTR mutation also activates the UPR, which, in turn, reprograms macrophages and is associated with an amplified inflammatory response in CF [54]. However, ROS can induce mutations in tumor suppressor genes like TP53 and activate oncogenic pathways such as EGFR, which are frequently altered in LC [55]. Additionally, CF-related oxidative stress can cause epigenetic modifications and impair DNA repair, further increasing the risk of oncogenic mutations [56]. These mechanisms suggest a potential link between CF and an elevated risk of carcinogenesis, particularly in the lungs.

Moreover, elevated levels of IL-1, IL-6, IL-8, IL-17, IL-33, granulocyte-macrophage colony-stimulating factor (GM-CSF), granulocyte colony-stimulating factor (G-CSF), and TNFα have been observed in the lungs and airways of patients of CF patients [57]. However, in a 20-year observational study of CF patients, Maisonneuve et al. identified a higher risk of developing cancers, especially in the reproductive organs, kidneys, and digestive system, including esophageal, gastric, bowel, and liver cancers, following organ transplantation [58]. Nevertheless, the immunosuppressive therapy used to treat the transplant can increase the risk of malignancy.

## 3. Chronic Lung Infections in Cystic Fibrosis

In CF, the worsening of lung function is linked with chronic colonization by pathogens. However, in CF airways, the level of inflammation seems to be excessive compared to the extent of the infection, characterized by significant infiltration of polymorphonuclear neutrophils (PMNs) and the release of pro-inflammatory molecules [59]. Macrophages play a crucial role in immune defense by both initiating inflammation and resolving it, helping to restore tissue balance [41]. There are two main types of macrophages: M1, which are pro-inflammatory and produce cytokines like TNFα, IL-1β, IL-6, and IL-12, and M2, which promote resolution of inflammation by secreting cytokines such as IL-10 and TGFβ1 [60]. In CF, the macrophage function is altered, contributing to persistent inflammation in the airways. CF macrophages are overactive, producing excessive pro-inflammatory cytokines in response to bacterial stimuli but are less effective at clearing bacteria [61]. It is known that *Staphylococcus aureus* (Sa) and *Pseudomonas aeruginosa* (Pa) species are major pathogens of CF lung disease, and their presence correlates with poor prognosis [62]. In particular, *Pa* is the most common bacterial species involved in the thickened mucus of the CF lung, triggering persistent immune responses. These immune cells release pro-inflammatory cytokines and ROS, contributing to oxidative damage, tissue remodeling, and a microenvironment that may facilitate carcinogenesis [63]. Moreover, methicillin-resistant *Staphylococcus aureus* (MRSA) is an early colonizer, worsening pulmonary decline and systemic inflammation, exacerbating cellular stress and DNA damage [64]. Additionally, anaerobic bacteria like *Prevotella* and *Fusobacterium*, though traditionally overlooked, produce inflammatory metabolites that further fuel chronic inflammation [65]. Together, these bacteria maintain a cycle of inflammation, oxidative stress, and impaired epithelial repair, accelerating lung damage and increasing the risk of carcinogenesis.

However, an analysis of prescription trends reveals an increase in the use of anti-pseudomonal nebulized antibiotics. This indicates a more aggressive approach to treating Pa over time. However, the use of prophylactic anti-staphylococcal antibiotics does not appear to be linked to a rise in Pa or an increase in the prevalence of other less common respiratory pathogens [66]. In addition, the treatment of lung infection in CF patients is limited by the presence of bacterial biofilms which provide protection against high levels of antimicrobials. These biofilms, formed by microorganisms in a self-secreted protein matrix, create a physically shielded environment and consist of diverse bacterial populations with varying metabolic and phenotypic characteristics [67]. Furthermore, Majka et al. investigated the relationship between advanced CF lung disease and sputum inflammatory mediators, as well as biofilm-forming bacteria. While no direct link was found between FEV1% and total sputum bacteria, high biofilm-forming bacteria correlated with more severe lung dysfunction. The study suggests that sputum, containing key biomarkers like IL-6, IL-8, IL-10, and elastase, could replace more invasive tests like BALF for monitoring CF disease progression [68]. Chronic respiratory infections in CF patients are mainly caused by opportunistic pathogens which can lead to respiratory failure. Analysis of CF patients’ sputum samples reveals that the pathogens involved are distinct from other medical conditions [69].

### Infections as Drivers of Inflammation and Tumorigenesis

Chronic infections can induce prolonged immune responses that create a pro-inflammatory environment. Around 20% of cancer cases are associated with chronic infections; while the immune system typically eliminates pathogens like viruses and bacteria quickly, certain tumorigenic pathogens manage to evade immune defenses, resulting in persistent infections that promote chronic inflammation and contribute to tumor development [70]. Lung macrophages play an important role in the tumor microenvironment, often promoting immunosuppression. As a result, chronic inflammation driven by macrophage-mediated inflammatory responses can serve as a precursor to the development of cancer [71]. Macrophages within tumors exist in distinct subsets, each capable of adopting a variety of phenotypes based on their local environment. The plasticity of macrophages is widely recognized, as they can sense, respond, and quickly adjust to changes in their surroundings to maintain tissue integrity and self-tolerance. However, when exposed to chronic challenges such as persistent irritant particles or infections, macrophages can shift to a more harmful role. In this state, they contribute to a sustained low-grade inflammatory response that may drive disease progression and potentially initiate cancer [72].

## 4. Dysbiosis of the Lung Microbiome in Cystic Fibrosis

### 4.1. Microbiome Composition in Cystic Fibrosis

It is well established that lung microbiome composition in CF patients differs significantly from healthy individuals [73]. Moreover, studies have shown that the microbiome composition in BALF of CF patients during the first year of life differs from that in oropharyngeal and nasopharyngeal samples [74]. This suggests that the microbiome diversity may change between the right and left lungs, as well as within different areas on the same side. By analyzing the microbiome in young CF patients, some diversity is maintained and Streptococcus is the most prevalent taxon [75]. However, as patients age, and with the extensive use of antibiotics, microbiome diversity decreases, with Pa and Burkholderia becoming more dominant, correlating also with the progression of lung function decline [76]. Cuthbertson et al. explored the relationship between the loss in microbiota diversity and lung function in CF patients, suggesting that microbiota could be used as an indicator of disease progression. By collecting sputum samples, the study revealed that in patients with severe disease, the core microbiota composition became more consistent, with Pa dominating as lung function deteriorated. In contrast, patients with better lung function had a higher prevalence of obligate anaerobes. Antibiotic use and FEV1% were the main factors influencing microbiota changes [77]. By integrating microbiota data with lung function analysis, this approach could improve the monitoring of disease progression in CF patients and help guide more personalized treatment strategies. Another significant implication of microbiome diversity is its potential link to exacerbations, which are characterized by worsening respiratory symptoms. These exacerbations may be associated with an increase in CF pathogens and a decrease in microbial diversity [78]. An approach that incorporates metagenomic analysis (to assess the functional potential of the microbiome), meta-transcriptomic analysis (to examine gene expression), meta-proteomics (to investigate catalytic functions), and metabolomics (to analyze metabolic activity) would allow us to gain a more comprehensive understanding of microbiome composition and its correlation with disease severity and exacerbations [79]. Identifying metabolites and gaining a deeper understanding of how metabolic activity evolves throughout disease progression is essential for improving clinical practices in managing CF. Furthermore, it is critical to determine whether CFTR modulators directly influence the lung microbiome in CF patients, as this knowledge could lead to more personalized treatment plans.

### 4.2. Impact of Dysbiosis on Inflammation and Cancer Development

**Figure 1:**
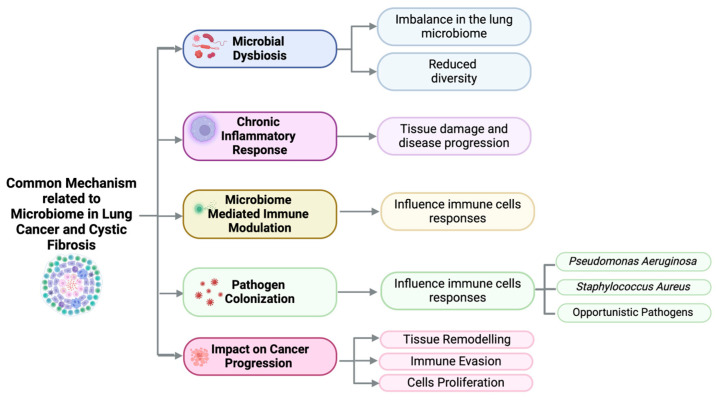
Common mechanisms related to microbiome in lung cancer and cystic fibrosis.

Recent studies have shown that lung microbiota in cancer patients differs significantly from healthy individuals, with enrichment of certain bacterial genera like *Granulicatella* and Streptococcus, and a decrease in diversity. The composition of the lung microbiota has been linked to chronic inflammation and cancer development, suggesting a possible role in LC progression [80]. It is important to emphasize that lung microbiota composition is influenced by lifestyle, pollution, and smoking. Some authors have suggested that an altered lung microbiome and chronic inflammation in lung tissue may contribute to carcinogenesis. The common mechanisms related to the microbiome in LC and CF are described in Figure 1. Decreased bacterial load in LC is linked to lower T-reg cell levels and enhanced recruitment of helper T-cells and NK cells, which reduces metastasis. Additionally, the lung microbiota can promote angiogenesis, a key factor in disease progression, through the activation of inflammatory mediators and increased VEGF expression [81]. Moreover, Helicobacter pylori (Hp) has been linked to tumor vascularization in LC through chronic inflammation and direct damage. The bacterium induces a systemic immune response and can promote lung cancer development. Hp’s VacA exotoxin, found in the lung, stimulates the production of interleukins IL-8 and IL-6 in lung cells, contributing to tumor angiogenesis. IL-8, in particular, is a key factor in promoting the formation of blood vessels in tumors [82]. The composition of microbiota can influence the effectiveness and side effects of cancer treatment. Gut dysbiosis is involved in carcinogenesis and the progression of LC through genotoxicity, systemic inflammation, and defective immunosurveillance [83]. Studies have shown that antibiotic-induced dysbiosis accelerates cancer progression, such as LC, by reducing immune response, including lower TNF-α levels and impaired leukocyte trafficking, leading to fewer activated CD8+ T cells in tumors [84]. Additionally, gut dysbiosis causes cellular immunosuppression in the lung, reducing key immune cells like macrophages and NK cells, and affecting T cell populations, further compromising pulmonary immunity. In some cases, antibiotic-induced gut microbiota imbalance may reduce the clinical effectiveness of immune checkpoint inhibitors (ICIs) in LC, with pre-treatment antibiotic use linked to a significant decrease in survival [85].

## 5. The Role of Oxidative Stress in Cystic Fibrosis

The immune response to infections results in the production of ROSs by immune cells such as neutrophils and macrophages. Indeed, inflammatory cells are sequestered in the pulmonary microvasculature and recruited into the airspaces following the generation of mediators such as IL-8, IL-1 and TNF-α in response to oxidative/nitrosative stress. The release of cytokines/chemokines induces neutrophil recruitment and activation of key transcription factors such as nuclear factor-kB (NF-kB) and antigen-activating protein-1 (AP-1) [86,87]. Moreover, oxidative stress impairs the function of antioxidant defense mechanisms, further amplifying cellular damage. This continuous cycle of inflammation, oxidative stress, and immune response damages lung tissue, impairs lung function, and accelerates disease progression in CF patients. Glutathione (GSH) is an important protective antioxidant against free radicals and other oxidants and has been implicated in immune modulation and inflammatory responses [86,88]. In particular, GSH is critical for the antioxidant defenses of the lungs, particularly in protecting the airspace epithelium from free radical- or oxidative-mediated injury and inflammation [89]. Alterations in GSH levels in lung lining fluid have been observed in various inflammatory conditions. GSH levels are decreased in the epithelial lining fluid (ELF) in CF patients [86]. Low GSH concentration in the ELF may contribute to an imbalance between oxidants and antioxidants in the lungs and may amplify inflammatory responses and potentiate lung injury [89]. Furthermore, activator protein-1 (AP-1) and AP-1-like antioxidant response element (ARE) have also been reported to modulate the expression of c-glutamylcysteine synthase (c-GCS), the rate-limiting enzyme in de novo GSH synthesis. It is possible that differences in ELF glutathione levels in various inflammatory lung diseases occur due to changes in the molecular regulation of GSH synthesis by AP-1 and the activation of ARE by oxidants and inflammatory and anti-inflammatory agents, its turnover/degradation and/or transport into lung cells [89]. In addition, the activation of neutrophils can oxidize and deactivate glutathione. However, GSH is quickly oxidized when it interacts with activated neutrophils, primarily due to the action of hypochlorous acid produced by myeloperoxidase (MPO) [90]. Previous studies suggest that CFTR is responsible for glutathione transport, which could explain the reduced GSH levels observed in the airways of patients with cystic fibrosis [73]. The steep oxygen gradient, in combination with the lack of nutrients like iron and zinc, are selective pressures toward strains that cause chronic *P. aeruginosa* infection [45,91]. Chronic infections have been shown to decrease in the last decade thanks to the use of new therapies with modulators and correctors of the CFTR protein. A study by Hisert et al. has demonstrated improvement in inflammation markers (NE, arginase-1, myeloperoxidase, calprotectin, IL1-β, and IL8) within a week of ivacaftor initiation [92]. Nevertheless, the dysfunction in the Nrf2 pathway, which regulates key antioxidant and detoxification genes, worsens the antioxidant response. In CF patients, insufficient activation of the HO-1/carbon monoxide pathway impairs immune function and bacterial clearance, leading to excessive inflammation [93]. The alteration of lipid metabolism is another mechanism connecting CFTR deficiency to oxidative stress; recent research has shown that CF airway pathology is associated with changes in fatty acids, ceramides, and cholesterol levels [94]. Indeed, elevated ceramide levels, sphingosine phosphate dysregulation, and impaired lipid degradation are closely associated with inflammation, oxidative stress, and dysfunctional autophagy in CF. Targeting lipid synthesis, such as myriocin, an inhibitor of sphingolipids synthesis, has been used to decrease inflammation, restore fatty acid metabolism, and improve pathogen clearance in CF [95].

## 6. Interactions Between Inflammation, Oxidative Stress, and Microbiome in Lung Cancer Development

**Figure 2:**
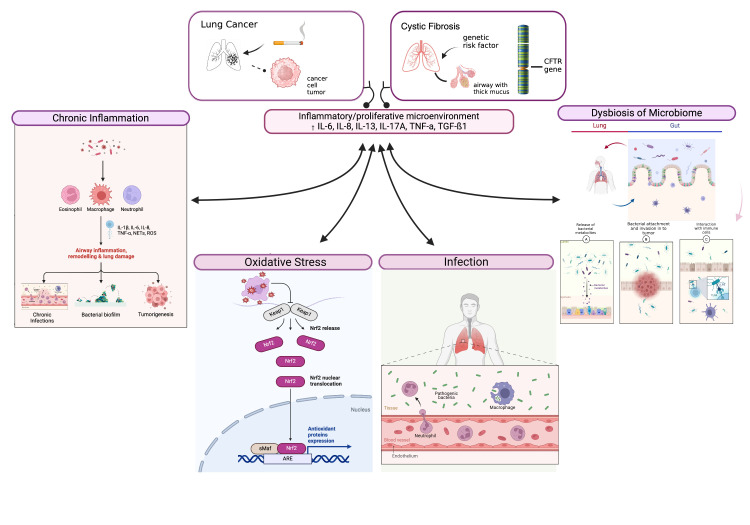
Interactions between chronic inflammation, oxidative stress, microbial dysbiosis and lung cancer development. The figure describes how chronic inflammation, oxidative stress and microbial dysbiosis interact to drive lung cancer development. These factors contribute to tissue damage, DNA mutations, and immune system regulation, fostering an environment for tumor initiation and progression.

Inflammation is a self-amplifying process, leading to chronic challenges to the integrity of airway epithelial cells that are responsible for the development and progression of LC [96]. It is often triggered by persistent infections or environmental factors, with the production of pro-inflammatory cytokines and ROS, which can cause cellular damage and mutations over time. Moreover, the accumulation of thick mucus impairs the function of ciliary mucus clearance, facilitating the colonization of bacteria and leading to infections, inflammation, and other complications. Oxidative stress plays a central role in amplifying cellular damage and hinders airway remodeling [97]. ROS not only damages cellular components such as lipids, proteins, and DNA but also disrupts the homeostasis of the lung environment, fostering an environment conducive to cancer development. In addition, the lung microbiome can either support or inhibit cancer development. A balanced microbiome contributes to immune system regulation and epithelial integrity, potentially offering protective effects [98]. However, microbial dysbiosis can create a pro-inflammatory and immunosuppressive environment that promotes tumor growth [99]. The interplay between these factors fosters a microenvironment conducive to tumor initiation and progression, highlighting the complex relationship between inflammation, oxidative stress, and microbial dysbiosis in LC development. The microbial changes could reflect changes in the LC environment to which the microbiome is responding. Identifying specific changes in the microbiome could open new possibilities for early detection, prognosis, and targeted therapeutic interventions (Figure 2). Moreover in Table 1 we have summarized the key molecular and pathogenic mechanisms linking CF and LC.

## 7. Clinical Implications

The association between CF and LC underscores the importance of increased awareness in clinical monitoring and the implementation of early detection strategies for CF patients. Given the role of chronic inflammation and microbial dysbiosis in tumor progression, routine screening for LC in CF patients should be integrated into clinical care, especially as life expectancy improves with new therapies. Additionally, therapeutic interventions aimed at reducing inflammation, restoring microbiome balance, and mitigating oxidative stress could play a critical role in cancer prevention and management.

The dysregulation of immune responses in CF patients is well known but it differs from that observed in the general population [45]. However, infections in CF remain a major cause of morbidity and mortality. The ARREST study showed that even after clearing *Pa*, inflammation can persist. Ongoing neutrophil elastase activity was linked to high IL-8 and neutrophil levels, suggesting that managing inflammation may help slow lung disease progression [100,101]. The treatment of CF remains complex, and recent advances in therapies such as CFTR correctors and modulators have dramatically transformed the therapeutic landscape of CF, shifting the focus from symptomatic management to targeted therapies that address the underlying genetic defects [102]. CFTR modulators improve CF symptoms by targeting the CFTR protein, with different types—potentiators, correctors and amplifiers—acting at various stage of its life cycle [103,104]. For instance, Ivacaftor, a CFTR potentiator, has shown significant clinical benefits for patients with specific CFTR gating mutations like G551D [102]. Elexacaftor, a CFTR corrector, in combination with tezacaftor and ivacaftor represents a major advancement in CF treatment, particularly for F508del mutations [105]. Clinical studies have shown significant improvements in lung function, represented by significant increases in FEV1, reductions in pulmonary exacerbations, and overall improvements in health-related quality of life. Understanding these molecular mechanisms is crucial for unraveling the link between CF and cancer risk [13]. At present, the available data on the long-term effects of modulator therapies on cancer risk in CF patients are limited due to the relatively recent introduction of these drugs and the need for long-term follow-up studies.

Future studies should investigate the potential of targeted treatments, such as CFTR modulators and microbiome-modulating therapies, to reduce the cancer risk in this population. Personalized care plans, incorporating both CF management and cancer prevention, could improve outcomes for CF patients at risk for LC. It is important to note that the search for predictive factors of treatment response and the management of LC must be personalized [106] and assessed on a case-by-case basis, also taking into account real-world evidence [107,108]. Recent data suggest that specific and locally administered probiotics in the respiratory tract might be a more targeted approach to prevent pathogen outgrowth in the lower airways; in particular, specific commensal bacteria in the airway microbiome mat protect against pathogen colonization [109]. However, further studies are needed to evaluate the safety and effectiveness of these taxa as live biotherapeutic products [110]. Notably, life expectancy of individuals with CF has significantly improved in recent decades, largely due to improvements in early diagnosis, multidisciplinary clinical care, and the development of CFTR modulator therapies. As a result, CF is no longer confined to childhood, and an increasing number of patients are living well into adulthood. This demographic shift brings new clinical challenges, including the potential for long-term complications such as malignancies. As evidenced by recent publication, a 35-year-old man with stable cystic fibrosis, not on CFTR modulators, was found to have ALK-translocated lung cancer after presenting with an assumed bacterial exacerbation. Treatment with the ALK inhibitor alectinib initially worsened his respiratory status, but adding CFTR-modulator therapy led to rapid clinical improvement [111]. This first reported case of ALK-positive lung cancer in a CF patient highlights the need to consider malignancy in atypical exacerbations and to be mindful of potential interactions between ALK inhibitors and CFTR function.

## 8. Conclusions and Future Perspectives

CF represents a significant challenge in understanding the complex mechanisms connecting chronic lung disease with an elevated risk of LC. Chronic inflammation, oxidative stress, and microbiome dysbiosis contribute to a pro-carcinogenic environment in CF lungs, potentially promoting tumor initiation and progression. As CF treatments continue to improve, increasing patient survival rates may elevate cancer risks, especially in the lungs. Therefore, further research into the molecular pathways linking CF and LC, along with the development of innovative clinical approaches, is essential to improve outcomes for CF patients. Early detection, close monitoring, and targeted therapies focusing on inflammation, microbial dysbiosis, and oxidative stress, could significantly decrease the risk of LC in this population.

Furthermore, significant advances in precision medicine open a new scenario of therapeutic possibilities. Gene editing tools, such as CRISPR targeting the CFTR gene, have the potential to correct underlying genetic defects and modify disease progression. In addition, early cancer detection methods using liquid biopsies and microbiome-derived biomarkers could revolutionize surveillance, enabling timely intervention before malignancy develops. Establishing comprehensive surveillance guidelines will support the clinician in creating personalized monitoring strategies.

## Figures and Tables

**Table 1:** Key mechanisms linking CF and LC.

Pathway/Mediator	Role in Cystic Fibrosis	Contribution to Lung Cancer
Chronic Inflammation	Persistent neutrophilic inflammation due to CFTR dysfunction and infections	Promotes epithelial-mesenchymal transition, DNA damage, angiogenesis, and tumor progression
Pro-inflammatory Cytokines	Elevated IL-6, IL-8, TNF-α, IL-1β in CF airways	Activate STAT3 and NF-κB pathways, enhancing cell proliferation and survival
Epigenetic Modifications	DNA methylation and histone changes in immune cells	Promote pro-tumorigenic immune phenotypes and chronic inflammation
Oxidative Stress (ROS)	Excess ROS from activated neutrophils and impaired antioxidant response (e.g., reduced glutathione)	Causes DNA damage, supports tumorigenesis, and disrupts epithelial integrity
Microbiome Dysbiosis	Loss of diversity, dominance of *P. aeruginosa* and *Burkholderia*, antibiotic overuse	Alters immune responses, sustains chronic inflammation, and may directly promote carcinogenesis
UPR (Unfolded Protein Response)	Activated by F508del-CFTR mutation and ER stress	Enhances macrophage inflammation and may promote survival of transformed cells
EGFR Pathway Activation	Upregulated in CF airway epithelium	Contributes to cell proliferation and tumor development
IL-10 / Treg Imbalance	Dysregulation contributes to unbalanced immune responses	Supports tumor immune evasion and progression
CFTR Modulator Effects	May modulate inflammation and microbiome composition	Potential indirect role in reducing LC risk (needs further research)

## Data Availability

Not applicable.
